# Acknowledgement to Reviewers of *Life* in 2014

**DOI:** 10.3390/life5010127

**Published:** 2015-01-07

**Authors:** Life Editorial Office

**Affiliations:** MDPI AG, Klybeckstrasse 64, CH-4057 Basel, Switzerland

The editors of *Life* would like to express their sincere gratitude to the following reviewers for assessing manuscripts in 2014:

Adamatzky, Andrew
Albers, Sonja-Verena
Aldersley, Michael
Alkorta, Itziar
Amato, Pierre
Anken, Ralf
Aou, Shuji
Arends, Karsten
Armstrong, Rachel
Austin, Daniel E.
Azevedo, Ana M.
Baba, Otto
Banerji, Anirban
Barbieri, Roberto
Barrangou, Rodolphe
Battchikova, Natalia
Baxter, Bonnie
Bernhardt, Harold S.
Bershad, Eric M.
Bibby, Thomas S.
Bich, Leonardo
Blankenship, Robert
Bläsi, Udo
Boomer, Sarah
Boto, Luis
Böttger, U.
Bowling, Lee
Braakman, Rogier
Brack, Andre
Braun, Dieter
Braun, Volkmar
Bredehöft, Jan
Brown, Aidan I.
Buckel, Wolfgang
Buratovich, Michael
Burrows, Lori L.
Caetano-Anolles, Gustavo
Carleton, Michael
Carter, Charles
Casamatta, Dale
Castro, Sarah
Chang, T.M.
Chao, Lin
Chauvat, Franck
Chen, Chien-Yu
Chen, Irene A.
Christos, Georgiou
Chteinberg, Serguei
Clarke, Jonathan D.A.
Cocca, Ennio
Conte-Camerino, Diana
Csonka, Laszlo
da Porto, Carla
Dabert, Patrick
Dade-Robertson, Martyn
Deamer, David
Deymier-Black, Alix C.
Di Giulio, Massimo
Dittmann, Elke
Dorn, Ronald I.
Durston, Kirk
Dvořák, Petr
Egel, Richard
Ehira, Shigeki
Eller, Fred
Fabris, Francesco
Ferlazzo, Fabio
Fernandez, Emilio
Filée, Jonathan
Foray, Nicolas
Forchhammer, Karl
Foster, Andrew W.
Foster, Jamie S.
Fox, George
Fuente, Vicenta
Fujita, Yuichi
Ganf, George
Geng, Yong-Jian
Gil, Rosario
Golden, James
Gonzalez, Juan
Gophna, Uri
Görlich, Dennis
Goswami, Nandu
Griffith, Robert W.
Grimm, Bernhardt
Gugger, Muriel
Hagemann, Martin
Hanke, Wolfgang R. L.
Harrison, Rene
Hauber, E.
Head, James
Hecht, Michael H.
Hellingwerf, Klaas J.
Helm, Richard
Herrero, Antonia
Hihara, Yukako
Hoiczyk, Egbert
Horn, E.
Horneck, Gerda
Hou, Harvey J. M.
Hubera, Harald
Ikehara, K.
Itoh, Itoh
Johnson, Phyllis J.
Kaplan, Aaron
Kasting, James
Kato, Souichiro
Keren, Nir
Kim, Jung-Rack
Kiss, John
Knaff, David B.
Koksharova, Olga
Komárek, Jiří
Komatsu, Goro
Kuhlman, Kimberly
Kurmayer, Rainer
Kvíderová, Jana
Larkum, Anthony
Latifi, Amel
Lavrentovich, Oleg D.
Lean, Oliver M.
Legue, Valerie
Lehman, Niles
Leigh, John
Leitner, Johannes
Luisi, Ben
Luisi, Pier Luigi
Luque Romero, Ignacio
Lutkenhaus, Joe
Ma, Kesen
Macias, Brandon R.
Madhusudhan, Nikku
Marco, Antonio
Markovitch, Omer
Martin, William
Matthijs, Hans
Mayer, Christian
McGowan, Eileen M.
McIntosh, Professor Andy
McKay, Chris
McKay, Christopher P.
Medina, Francisco Javier
Melott, Adrian
Mishra, Arun K.
Moeller, Ralf
Monnard, Pierre-Alain
Moroder, Luis
Mueller, Sabine
Mullineaux, Conrad W.
Nagai, Hiroki
Nagaraja, Mamta Patel
Nixdorf, Brigitte
Noireaux, Vincent
Noller, Harry F.
O’Brien, Edward
O'Donoghue, Patrick
Olasagasti, Felix
Oren, Aharon
Otero, José M.
Paerl, Hans W.
Patil, Avinash
Pennock, Robert T.
Penny, David
Pentecost, Allan
Pereira, Sara Isabel
Perona, John J.
Perrin, David
Petroff, Alex
Ploux, Olivier
Prangishvili, David
Rai, Lal Chand
Reis, Pedro Miguel Ramos
Rettberg, Petra
Richardson, Laurie
Risser, Douglas D.
Robertson, Deb
Robinson, Tom
Rohde, Christine
Russell, Michael J.
Ruyters, G.
Rzymski, Piotr
Salmaso, Nico
Sato, Naoki
Sato, Yui
Schaefer, Laura
Scherer, Günther
Schild, Rudolf E.
Semenov, Dmitry
Serra, Roberto
Sheldon, Robert B.
Sherman, Louis A.
Shirt-Ediss, Ben
Siksnys, Virginijus
Smith, Leslie S.
Stan-Lotter, Helga
Stano, Pasquale
Steinberg, Susan
Stern, Jennifer C.
Stoker, Carol R.
Strazewski, Peter
Suedfeld, Peter
Syvanen, Michael
Szewczyk, Nathaniel J.
Tajmir-Riahi, H. A.
Takabe, Teruhiro
Tessera, Marc
Theil, Marco
Thompson, William R.
Trifonov, Edward
Tyystjärvi, Taina
Unrau, Peter
Vanwonterghem, Inka
Vass, Imre
Ventura, Stefano
Vicente, Miguel
Vicuña, Rafael
Villani, Marco
Vioque, Agustín
Visser, Petra
Volkmann, Dieter
Voß, Björn
Walde, Peter
Waldron, Kevin
Westby, Chris
Whitton, Brian A.
Wickramasinghe, Chandra
Wilde, Annegret
Williams, Stan
Yarus, Michael
Yomo, Tetsuya
Yonath, Ada E.
Yoshikawa, Kenichi
Zhaxybayeva, Olga

